# Simultaneous fingerprint and high-wavenumber fiber-optic Raman spectroscopy improves *in vivo* diagnosis of esophageal squamous cell carcinoma at endoscopy

**DOI:** 10.1038/srep12957

**Published:** 2015-08-05

**Authors:** Jianfeng Wang, Kan Lin, Wei Zheng, Khek Yu Ho, Ming Teh, Khay Guan Yeoh, Zhiwei Huang

**Affiliations:** 1 Optical Bioimaging Laboratory, Department of Biomedical Engineering, Faculty of Engineering, National University of Singapore, Singapore 117576; 2 Department of Medicine, Yong Loo Lin School of Medicine, National University of Singapore and National University Health System, Singapore 119260; 3 Department of Pathology, Yong Loo Lin School of Medicine, National University of Singapore and National University Health System, Singapore 119074.

## Abstract

This work aims to evaluate clinical value of a fiber-optic Raman spectroscopy technique developed for *in vivo* diagnosis of esophageal squamous cell carcinoma (ESCC) during clinical endoscopy. We have developed a rapid fiber-optic Raman endoscopic system capable of simultaneously acquiring both fingerprint (FP)(800–1800 cm^−1^) and high-wavenumber (HW)(2800–3600 cm^−1^) Raman spectra from esophageal tissue *in vivo*. A total of 1172 *in vivo* FP/HW Raman spectra were acquired from 48 esophageal patients undergoing endoscopic examination. The total Raman dataset was split into two parts: 80% for training; while 20% for testing. Partial least squares-discriminant analysis (PLS-DA) and leave-one patient-out, cross validation (LOPCV) were implemented on training dataset to develop diagnostic algorithms for tissue classification. PLS-DA-LOPCV shows that simultaneous FP/HW Raman spectroscopy on training dataset provides a diagnostic sensitivity of 97.0% and specificity of 97.4% for ESCC classification. Further, the diagnostic algorithm applied to the independent testing dataset based on simultaneous FP/HW Raman technique gives a predictive diagnostic sensitivity of 92.7% and specificity of 93.6% for ESCC identification, which is superior to either FP or HW Raman technique alone. This work demonstrates that the simultaneous FP/HW fiber-optic Raman spectroscopy technique improves real-time *in vivo* diagnosis of esophageal neoplasia at endoscopy.

Esophageal cancer is the eighth most frequent malignancy worldwide with a 5-year survival rate of approximately 19% in the Unites States^1^,^2^. Esophageal squamous cell carcinoma (ESCC) is one of the deadly esophageal cancers^3^. Current routine diagnosis of ESCC is based on white light reflectance (WLR) endoscopy which heavily relies on visual identification of gross morphological tissue changes, leading to poor diagnosis accuracy^1^,^2^,^3^. Existing diagnostic guidelines recommend extensive biopsy samplings (typically four-quadrant samplings) at every 1–2 cm interval along suspicious lesions segments during endoscopic inspections of patients^4^. This approach produces a vast number of negative biopsies without much improving the diagnostic yield, but is clinically labor intensive and a burden to the patients. There is a need to develop advanced optical diagnostic techniques for objective diagnosis and characterization of esophageal tissue with biomolecular specificity.

Optical spectroscopic techniques (e.g., fluorescence, diffuse reflectance, and Raman) have been comprehensively investigated for precancer and cancer diagnosis in internal organs (e.g., stomach, esophagus, colon, bladder, and lung, etc.)^5^,^6^,^7^,^8^,^9^,^10^,^11^,^12^,^13^,^14^,^15^,^16^,^17^,^18^,^19^,^20^,^21^,^22^,^23^,^24^,^25^. Raman spectroscopy is an optical vibrational technique based on the fundamental principle of inelastic light scattering^26^, which is capable of probing biochemical and biomolecular structures and conformations of cells and tissue associated with disease transformation^7^,^8^,^9^,^10^,^11^,^12^,^13^,^14^,^15^,^16^,^17^,^18^,^19^,^20^,^21^,^22^,^23^,^24^,^25^. Currently, biomedical Raman research in diagnosing precancer and cancer is mostly centered on the fingerprint (FP) range (i.e., 800–1800 cm^−1^) that contains rich biochemical information about the tissue^7^,^8^,^9^,^10^,^11^,^12^,^13^,^14^,^15^,^16^,^17^,^18^. The unrivaled advantage of the FP Raman spectroscopy technique stems from its capability to uncover specific information about backbone structures of proteins, lipids and nucleic acid assemblies in cells and tissue^7^,^8^,^9^,^10^,^11^,^12^,^13^,^14^,^15^,^16^,^17^,^18^. The efficiency of the FP Raman spectroscopy technique is, however, compromised in certain organ sites owing to extremely weak tissue Raman signals but overwhelming tissue autofluorescence (AF) background. Recent attentions have been directed towards the use of high-wavenumber (HW) regime (e.g., 2800–3600 cm^−1^), as the HW spectral range exhibits stronger tissue Raman signals with less AF interference^19^,^20^,^21^,^22^,^23^,^24^,^25^. The clinical rationales for combining the FP and HW spectral ranges for *in vivo* esohphageal Raman measurements are therefore manifold: (i) For tissues that could exhibit intense AF overwhelming the tissue FP Raman signals, the HW range could still contain intense tissue Raman peaks with diagnostic information, (ii) The FP and HW Raman spectra offer complementary biomolecular information, and the integrated FP/HW Raman technique could improve tissue characterization and diagnosis^24^,^25^. To date, the simultaneous FP and HW Raman spectroscopic technique has not yet been investigated for distinguishing ESCC from normal esophagus. This work aims to assess the clinical value of a simultaneous FP and HW fiber-optic Raman spectroscopy technique developed for improving real-time *in vivo* diagnosis of ESCC at endoscopy. Unpaired two-sided Student’s *t*-test is employed to extract the statistically different Raman spectral features between ESCC and normal esophageal tissues. Partial least squares - discriminant analysis (PLS-DA) and leave-one patient-out, cross validation (LOPCV) are further implemented to develop robust diagnostic algorithms for differentiation between ESCC and normal esophagus.

## Results

**Figure 1:**
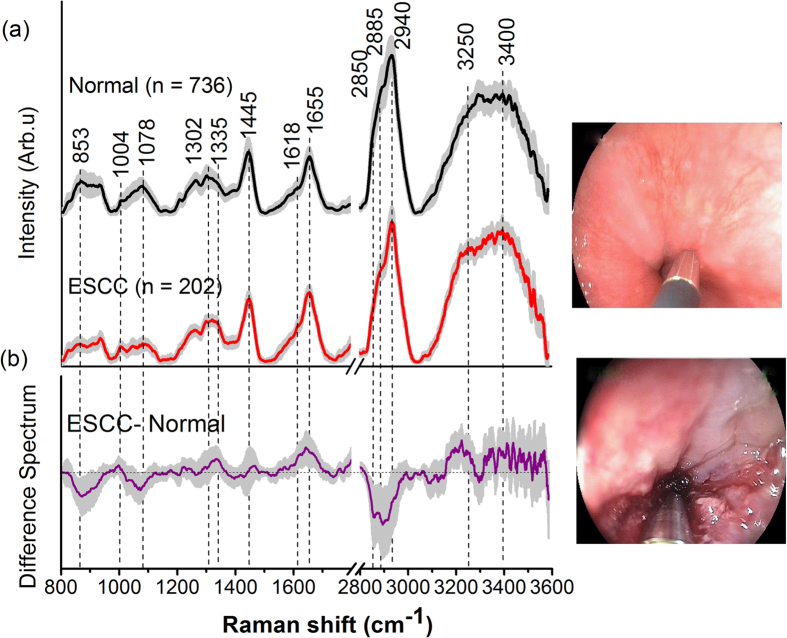
(**a**) The mean *in vivo* FP/HW Raman spectra ± 1 standard deviation (SD) of the training dataset (80% of the total dataset) (normal (n = 736); ESCC (n = 202)) for diagnostic algorithms development; (**b**) Difference spectra (ESCC - normal) ± 1 SD resolving the unique spectral features of ESCC. The corresponding images of the WLR-guided FP/HW Raman procedures on normal esophagus and ESCC are also shown.

A total of 1172 *in vivo* FP/HW tissue Raman spectra (normal (n = 860); ESCC (n = 312)) were acquired from 48 esophageal patients undergoing routine endoscopic examination (see Supplementary Fig. S1 online). The total *in vivo* Raman dataset acquired was split into two parts: i.e., 80% of the total dataset for training (938 *in vivo* FP/HW Raman spectra [normal (n = 736); ESCC (n = 202)] from 34 esophageal patients); while the remaining 20% of the total dataset for predictive testing (234 *in vivo* FP/HW Raman spectra [normal (n = 124); ESCC (n = 110)] from 14 esophageal patients). Figure 1(a) shows the mean *in vivo* FP/HW tissue Raman spectra ± 1standard deviation (SD) (shaded area) of the training dataset (80% of the total dataset) for tissue diagnostic algorithms development. The corresponding images of the WLR-guided FP/HW Raman procedures are also shown in Fig. 1. Prominent esophageal tissue Raman peaks with tentative assignments^7^,^8^,^9^,^10^,^11^,^12^,^13^,^14^,^15^,^16^,^17^,^18^ can be observed in the FP region, i.e., 853 (*v*(C-C) proteins), 1004 (ν_s_(C-C) ring breathing of phenylalanine), 1078 (*ν*(C-C) of lipids), 1265 (amide III v(C-N) and δ(N-H) of proteins), 1302 (CH_2_ twisting and wagging of lipids), 1335 (CH_3_CH_2_ twisting of proteins and nucleic acids), 1445 (δ(CH_2_) deformation of proteins and lipids), 1618 (v(C = C) of porphyrins), 1655 (amide I v(C = O) of proteins) and 1745 cm^−1^ (v(C = O) of phospholipids). Intense Raman peaks are also observed in the HW region^18^,^19^,^20^,^21^,^22^,^23^,^24^,^25^, i.e., 2850 and 2885 cm^−1^ (symmetric and asymmetric CH_2_ stretching of lipids), 2940 cm^−1^ (CH_3_ stretching of proteins), ~3300 cm^−1^ (amide A (NH stretching of proteins)) and the broad Raman band of water (OH stretching vibrations peaking at ~3250 and ~3400 cm^−1^) that are related to the local conformation and interactions of OH-bonds in the intracellular and extracellular space of esophageal tissue^27^,^28^. The intense broad Raman band of water above 3000 cm^−1^ has also been observed in other soft tissues (e.g., brain, oral tissue)^27^,^28^. Figure 1(b) shows the difference Raman spectra between ESCC and normal esophageal tissue ± 1SD (shaded area), reflecting the Raman-active component changes associated with cancerous progression in the esophagus. The significant difference (p = 1.3E-8, unpaired two-sided Student’s t-test) in Raman spectra of ESCC and normal tissue discerned (Fig. 1(b)) demonstrates the potential of simultaneous FP/HW Raman endoscopy for *in vivo* diagnosis of esophageal cancer.

**Figure 2:**
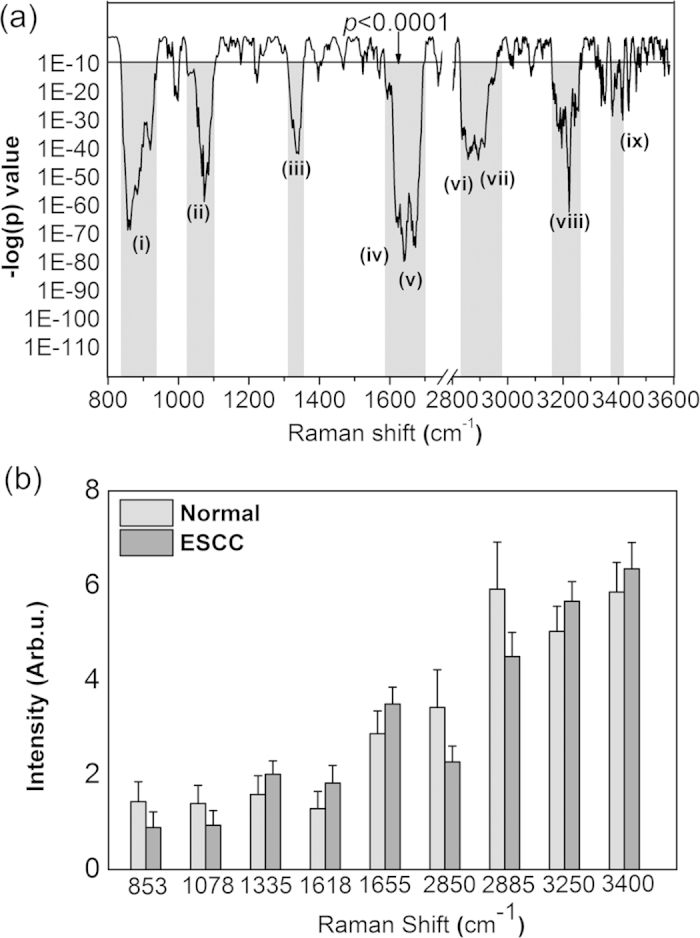
(**a**) Unpaired two-sided Student’s t-test on Raman peak intensities of the training dataset (80% of the total dataset) (normal (n = 736); ESCC (n = 202)) over the entire spectral range (i.e., 800–1800 cm^−1^ and 2800–3600 cm^−1^). Seven Raman spectra sub-regions containing the diagnostically significant information were identified. (b) Histogram ± 1 SD of the most diagnostically significant Raman peaks (*p < 1E-10).

**Figure 3:**
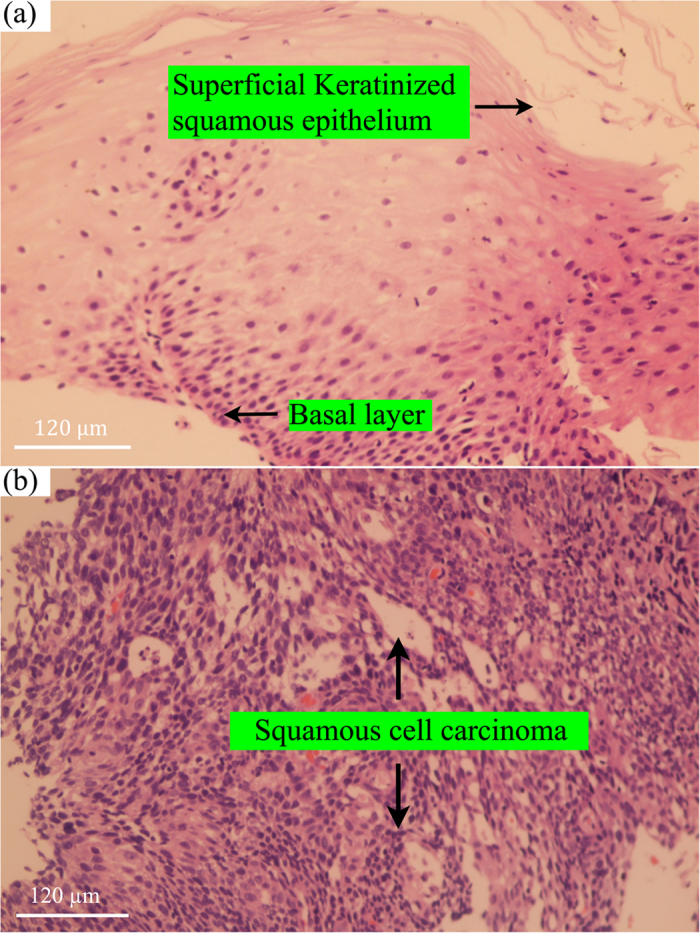
Representative hematoxylin and eosin (H&E)-stained histopathologic slides (original magnification, ×200) corresponding to different esophageal tissue types measured: (**a**) Normal superficial keratinised squamous epithelium and the basal layer; (**b**) Invasive esophageal squamous cell carcinoma showing prominent architectural and cytological atypia.

To elucidate the diagnostically important Raman-active components, Fig. 2(a) shows a logarithmic plot of the calculated *p*-values (unpaired two-sided Student’s t-test) for each of the Raman intensities in the entire spectral range (i.e., 800–1800 cm^−1^ and 2800–3600 cm^−1^). In particular, seven spectral sub-regions with statically significant difference (p < 1E-10) between ESCC and normal esophagus were found: i.e., 840–940 cm^−1^, 1025–1100 cm^−1^, 1310–1355 cm^−1^, 1585–1690 cm^−1^, and 2830–2975 cm^−1^ related to proteins, lipids and nucleic acids. Significant spectral differences were also observed in bound water in the ranges of 3160–3260 cm^−1^ and 3370–3420 cm^−1^. Figure 2(b) displays a histogram of the most statistically different Raman peak intensities (mean ± 1SD) for both the FP and HW ranges, i.e., (i) 853 cm^−1^, (ii) 1078 cm^−1^, (iii) 1335 cm^−1^, (iv) 1618 cm^−1^, (v) 1655 cm^−1^, (vi) 2850 cm^−1^, (vii) 2885 cm^−1^, (viii) 3250 cm^−1^, and (ix) 3400 cm^−1^. The histopathology identifies prominent cellular and architectural anomalies in ESCC (Fig. 3), while the relatively higher or lower FP/HW tissue Raman bands representing different Raman-active components reveal the specific biochemical/biomolecular changes of esophageal tissue accompanied with ESCC transformation. The changes of FP/HW Raman spectra related to lipids, proteins, DNA and water contents in tissue reconfirm the capability of simultaneous FP/HW Raman spectroscopy to detect ESCC at the molecular level.

**Figure 4:**
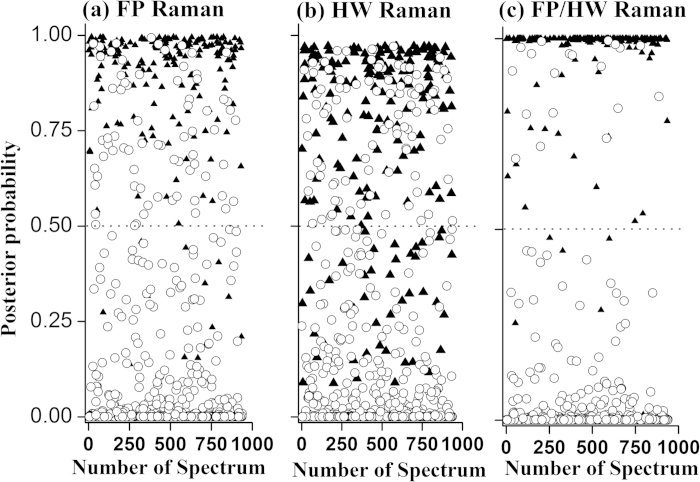
The posterior probabilities of *in vivo* Raman spectra belonging to (i) normal esophagus (n = 736), and (ii) ESCC (n = 202) of the training dataset (80% of the total dataset), using partial lease square-discriminant analysis and leave-one-patient out cross validation based on the FP, HW and the integrated FP/HW Raman techniques, respectively. (◯) normal; (▲) ESCC.

**Figure 5:**
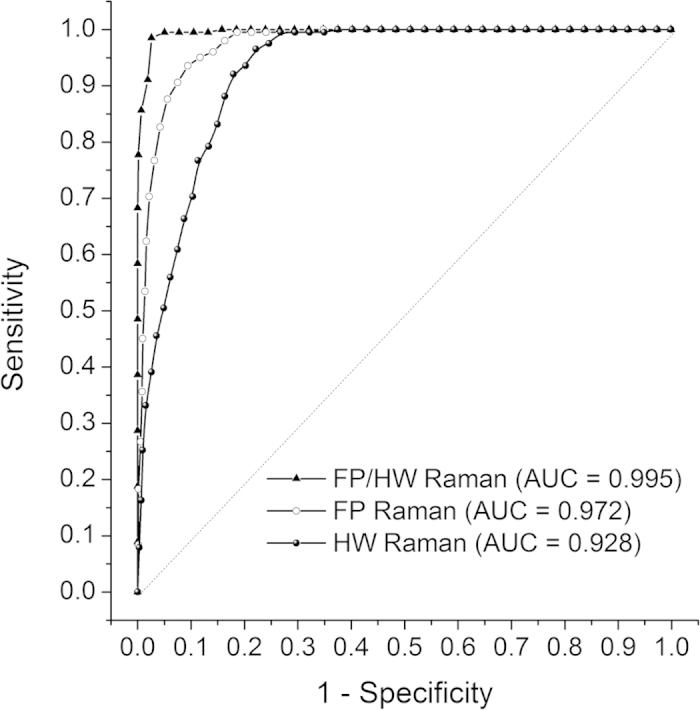
Receiver operating characteristic (ROC) curves for separating ESCC from normal esophageal tissue for the training dataset (80% of the total dataset). The areas under the ROC curves (AUC) are 0.972, 0.928 and 0.995, respectively, using the FP, HW and the integrated FP/HW Raman techniques.

Capitalizing on the complementary biochemical/biomolecular information identified in both the FP and HW spectral ranges, PLS-DA and LOPCV are implemented on the training dataset (80% of the total dataset) (Fig. 1(a)) to develop robust diagnostic model for enhancing *in vivo* ESCC diagnosis. A Cohen’s kappa of 0.91 demonstrated a high level of agreement between the independent pathologists for the esophageal tissue groupings^29^. Figure 4 shows the scattered plots of cross-validated PLS-DA posterior probability of each Raman prediction for (a) FP, (b) HW, and (c) integrated FP/HW, respectively. The diagnostic accuracy with integrated FP/HW Raman spectroscopy is 97.3% [sensitivity of 97.0% (196/202) and specificity of 97.4% (717/736)], superior to using either FP (accuracy of 90.9%, sensitivity of 93.6% (189/202), and specificity of 90.2% (664/736)) or HW (accuracy of 85.5%, sensitivity of 78.2%(158/202), and specificity of 87.5% (644/736)) Raman technique alone. The receiver operating characteristic (ROC) curves are also generated (Fig. 5), with the integration areas under the ROC curves of being 0.972, 0.928 and 0.995, respectively, for the FP, HW and the integrated FP/HW techniques. The above results confirm that the integrated FP/HW Raman technique provides the best diagnostic performance for *in vivo* ESCC detection as compared to FP or HW Raman technique.

In the light of the promising diagnostic results presented (Figs 4 and 5), we have applied the simultaneous FP/HW Raman spectroscopy and diagnostic algorithms developed for predictive diagnosis of the independent testing dataset (20% of the total dataset)(see Supplementary Fig. S2 online). The predictive accuracy of 93.2% (i.e., sensitivity of 92.7% (102/110) and specificity of 93.6% (116/124)) can be achieved by using the integrated FP/HW Raman spectroscopy, substantiating the advantages of simultaneous FP/HW Raman spectroscopy over FP (predictive accuracy of 91.0%; sensitivity of 90.9% (100/110), and specificity of 91.9% (113/124)) or HW (predictive accuracy of 80.3%; sensitivity of 76.4% (84/110), and specificity of 83.9% (104/124)) Raman technique for *in vivo* ESCC diagnosis (see Supplementary Fig. S3 online).

## Discussion

We have successfully acquired high-quality *in vivo* FP/HW Raman spectra from esophagus tissue in real-time (<1 sec) during clinical endoscopic examination. Significant FP and HW Raman spectral differences between ESCC and normal esophageal tissue are observed. Statistically significant different sub-regions of FP Raman bands (840–940, 1025–1100, 1310–1355, and 1585–1690 cm^−1^) were identified, uncovering highly specific biochemical changes of esophageal tissue associated with ESCC transformation. For instance, ESCC was characterised by significantly reduced Raman peak intensities at 1078 cm^−1^ (v(C = C)) (p = 2.3E-59), indicating a relative reduction in lipid content that is likely due to the thickening of the cancerous esophageal mucosa obscuring the Raman signals from deeper tissue layers^30^. The increase of proteins content was indicated by the sensitive biomarkers at 1618 cm^−1^ and the band broadening of 1655 cm^−1^ (amide I v(C = O) of proteins) for ESCC. This observation is in agreement with the molecular biology study whereby an over-expression of various proteins associated with ESCC has been reported^31^. Moreover, ESCC shows an increased DNA content exhibiting much stronger Raman intensity at 1335 cm^−1^ (CH_3_CH_2_ twisting of proteins and nucleic acids), which is complied with the DNA changes associated with precancer and cancer transformation^7^,^8^,^9^,^10^,^11^,^12^,^13^,^14^,^15^,^16^,^17^,^18^,^24^,^25^. On the other hand, unpaired two-sided Student’s t-test identified three statistically different sub-regions of HW Raman bands (2830-2975, 3160-3260 and 3370–3420 cm^−1^) related to lipids, proteins and water changes associated with ESCC transformation. ESCC was characterised by a relatively decreased lipid and increased protein content as indicated by the higher Raman peak intensity ratio of I_2940_/I_2850_in ESCC. The result is consistent with the changes of lipids and proteins content observed in other clinical studies of Raman spectroscopy for disease diagnosis^7^,^8^,^9^,^10^,^11^,^12^,^13^,^14^,^15^,^16^,^17^,^18^,^19^,^20^,^21^,^22^,^23^,^24^,^25^. Different water content (OH stretching vibrations peaking at ~3250 and ~3400 cm^−1^) was observed between ESCC and normal esophagus as indicated by the significantly different ratio (p = 3.2E-7) of asymmetric to symmetric OH stretching peaking at ~3250 and ~3400 cm^−1^, reflecting the local structure of hydrogen-bonded networks in the epithelium^32^. The relative increase of the OH stretching vibrational intensity suggests that neoplastic epithelium is accompanied with an increased content of water in tissue associated with ESCC, which is probably due to the increased cellularity and proliferation of ESCC in the esophagus^32^,^33^. Three Raman peak intensity ratios (i.e., I_2885_/I_2940_, I_2940_/I_3250_, and I_2885_/I_3250_) were also calculated, suggesting that ESCC could be separated from normal esophageal tissue with sensitivities of 48.5% (98/202), 57.9% (117/202), 73.3% (148/202), and specificities of 54.6% (402/736), 64.1% (472/736), 64.3% (473/736), respectively. These findings indicate that the water perfusion in ESCC is one of the important biomarkers for esophageal neoplasia detection *in vivo*.

To employ the Raman-based biochemical/biomolecular information for ESCC diagnosis, we further developed a robust PLS-DA classification model constructed from the training dataset (80% of the total dataset). The PLS-DA algorithms based on the integrated FP/HW Raman spectra could differentiate ESCC from normal esophagus mucosa with a good diagnostic accuracy (accuracy of 97.3% (913/938)). The FP Raman spectrum contains highly specific information about proteins, lipids and DNA conformations. On the other hand, the HW spectral range contains complementary information related to local conformation of bound and unbound water as well as CH_2_ and CH_3_ stretching moieties of lipids and proteins that are not reflected in the FP range, enabling back-tracking of misclassified Raman spectra. For instance, FP and HW Raman spectroscopy misclassified 85 and 136 spectra, respectively, from the 11 and 23 patients recruited, in which 9 misclassified patients were identical in both the modalities. The integrated FP/HW Raman technique reduced the number of misclassified patients to 4 (25 spectra). Hence, the complementary biochemical/biomolecular information harvested by the simultaneous FP/HW Raman spectroscopy could greatly improve the *in vivo* diagnosis of ESCC at endoscopy. Furthermore, misclassified spectra by the FP Raman (that were correctly classified by the integrated FP/HW Raman) are comprised of weaker tissue Raman signals and relatively high AF background; while the addition of the HW Raman containing stronger tissue Raman peaks (e.g., 2885, 2940, 3250 and 3400 cm^−1^) but much reduced AF could improve the overall signal-to-noise ratio (SNR) of integrated FP/HW Raman spectra, therefore, enhancing the performance of classification. In addition, prospective study on the independent testing dataset (20% of the total dataset) (see Supplementary Figs. S2-S3 online) shows that the integrated FP/HW Raman spectroscopy could provide a predication accuracy of 93.2% (218/234) for ESCC identification. The above results affirm that the integrated FP/HW Raman spectroscopy could be a potent optical diagnostics means to revealing comprehensive but complementary endogenous biomolecular information of epithelial tissue, thereby enhancing *in vivo* objective diagnosis of esophageal neoplasia.

The introduction of FP/HW Raman spectroscopy could have a major impact on current endoscopic practice and decision making by providing objective real-time diagnosis. Several clinical scenarios exist for the applications of FP/HW Raman spectroscopy during clinical endoscopic examinations. One scenario is for the screening and surveillance. FP/HW Raman spectroscopy can be used by the endoscopists as a guiding tool to reduce negative biopsy numbers by objectively evaluating wide lesion regions in real-time without extensive excisional biopsies of the tissues, reducing the risk and the cost of the procedure. Further, the individual patients having higher risk of developing ESCC could be identified by Raman spectroscopy for quick surveillance and examination at endoscopy. Therefore, FP/HW Raman spectroscopy is expected to provide a greater opportunity for curative treatment with improved patient outcome. In another scenario of interventional procedures, FP/HW Raman spectroscopy could be used to assist in determining the lesion margins during endoscopic mucosal resection (EMR) and endoscopic submucosal dissection (ESD) procedures for the treatment of ESCC^34^,^35^. The FP/HW Raman spectroscopy system developed is now routinely used by both experienced and novice endoscopists without any difficulty at Endoscope Center at the National University Health System, Singapore for screening esophageal patients for targeted biopsies. Considering its encouraging diagnostic performance (Fig. 4(c)), fiber-optic FP/HW Raman spectroscopy has promise to become one part of routine endoscopic practices. Currently, clinical trials on a larger series of esophageal patients based on the simultaneous FP/HW Raman spectroscopy technique are in progress to further assess its clinical merits for precancer and early cancer diagnosis in the esophagus.

In summary, we demonstrate that the simultaneous FP/HW *in vivo* Raman spectra of esophageal tissue can be acquired in real-time during clinical endoscopic examination. Significant FP/HW Raman spectral differences between ESCC and normal esophageal tissue are observed. The use of complementary biomolecular information harvested through the integrated FP/HW Raman spectroscopy significantly enhances the detection and diagnosis of esophageal neoplasia as compared to either the FP or HW Raman technique alone. This unique biomolecular endoscopic approach based on the simultaenous FP/HW Raman spectroscopy could open a new avenue for objective diagnosis of esophageal neoplasia *in vivo* at the molecular level.

## Methods

### Raman Instrumentation

The Raman fiber-optic simultaneous FP and HW spectroscopy platform developed for *in vivo* tissue Raman measurements at endoscopy consists of a 785 nm diode laser (maximum output: 300 mW, B&W TEK Inc.), a high-throughput reflective imaging spectrograph (Acton LS-785 f/2, Princeton Instruments Inc.) equipped with a customised 830 gr/mm gold-coated grating that has a ~85% diffraction efficiency in the NIR range of 800–1200 nm. A thermo electric-cooled (−70 °C), NIR-optimised deep-depletion charge-coupled device (CCD) camera (PIXIS 400BR-eXcelon, Princeton Instruments Inc.) was used to collect the FP/HW Raman signals. A customised parabolic-aligned fiber bundle (64 × 100 μm fibers, numerical aperture (NA) = 0.22) was coupled into the entrance slit to compensate for the image aberration in the broad spectral range (800–3600 cm^−1^)^17^,^36^, which significantly improves the signal-to-noise ratio (SNR) (20-fold improvement) as well as the spectral resolution (~9 cm^−1^) of the Raman system as compared to a conventional straight slit imaging spectrograph. A depth-selective bevelled fiber-optic Raman endoscopic probe (1.8 mm in outer diameter) compatible with most medical endoscopes was developed for *in vivo* tissue Raman measurements at endoscopy. The customised Raman endoscopic probe comprises central light delivery fiber (200 μmin in diameter, NA = 0.22) surrounded by 18 bevelled collection fibers (200 μm in diameter, NA = 0.22). A 1.0 mm sapphire ball lens (NA = 1.78) is further coupled to the fiber tip of the probe to focus the excitation light onto the tissue surface^37^. Monte Carlo simulation shows that ~85% of the total Raman signal collected by the bevelled fiber-optic Raman endoscopic probe is arising from the top ~300 μm esophageal layer with an estimated tissue probing volume of ~0.017 mm^3^; whereas ~15% of the Raman collection is from the stromal layer (~300-800) μm of the esophageal tissue with an estimated tissue probing volume of ~0.003 mm^3^, facilitating the *in vivo* detection of esophageal early cancer and precancer^37^.The 785-nm laser excitation power is ~12 mW on the tissue, which is less than the American National Standards Institute (ANSI) maximum permissible skin exposure limit set out for a 785-nm laser beam^38^. Further finite difference thermal modeling^39^,^40^ based on the optical properties of esophageal tissue^41^,^42^ shows that even without consideration of other cooling effects (e.g., perfusion and evaporation in tissue), the maximum tissue temperature rise is only about 0.07 °C after 1-min of 785-nm laser radiation with an incident power density of ~1.5 W/cm^2^ during tissue Raman measurements. This temperature rise estimated is far below the level to cause photothermal damage to tissue and cells^43^, suggesting that the laser power used in this study is safe for *in vivo* tissue Raman measurements. The system was wavelength-calibrated using a mercury/argon calibration lamp (HG-1 and AR-1, Ocean Optics Inc., Dunedin, FL) in the FP range, and 4-acetamidophenol that exhibits strong well-defined peaks in the HW region at 2931 cm^−1^ and 3064 cm^−1^ (ASTM E1840 standard) is used for the HW wavelength calibration. The spectral response correction for the wavelength-calibrated system was conducted using a standard lamp (RS-10, EG&G Gamma Scientific, San Diego, CA)^44^. The raw FP/HW Raman spectra measured from *in vivo* esophageal tissue represent a combination of weak tissue Raman scattering, intense AF background and the noise. The raw spectra are preprocessed by a third-order Savitzky-Golay smoothing filter (a window width of 3 pixels) to remove the spectral noise. In the FP region (800–1800 cm^−1^), a fifth-order polynomial was found to be optimal for fitting the AF background in the noise-smoothed spectrum and this polynomial was then subtracted from the measured FP spectrum to yield the FP tissue Raman spectrum alone. In the HW range (2800–3600 cm^−1^), a first-order polynomial fit was used for removing the AF background. The tissue FP/HW Raman spectrum was finally normalised to the integrated area of the entire FP and HW range, enabling a better comparison of the spectral shapes and relative Raman band intensities between ESCC and normal esophagus. All the preprocessing is completed within 0.5 sec, and the processed Raman spectra and diagnostic outcomes can be displayed on the computer screen in real-time by using the house-developed online Raman software^44^.

### Clinical trial protocol

The ethical protocol of the present study was approved by the Institutional Review Board (IRB) of the National Healthcare Group (NHG) of Singapore. Prior to Raman measurements, all patients (21 to 80 years old) signed an informed consent permitting *in vivo* Raman spectroscopic measurements during endoscope examinations. The trial has been registered (registration number: ISRCTN15587241) on June 23, 2015 into ISRCTN registry (http://www.isrctn.com/), which is a publicly accessible primary register that participates in the World Health Organization (WHO) International Clinical Trial Registry Platform. Also, the trial was performed in accordance with International Conference on Harmonization (ICH) for Good Clinical Practice (GCP) guidelines, Declaration of Helsinki (2000). A total of 48 esophageal patients (31 men and 17 women with mean ages of 51) were enrolled in the gastrointestinal Raman endoscopic examinations for various esophageal indications (e.g., anemia, bleeding, eating problems, pain etc.). One notes that the patients presenting with co-morbid diseases, severe acute/chronic medical conditions or bleeding disorders, in which biopsies may be contraindicated were excluded from this study. During endoscopic examination of suspicious lesions, the Raman probe was placed in gentle contact with the esophageal mucosa surface, and the positioning of the Raman probe against the tissue sites was verified on the computer monitor by the endoscopists in-charge at endoscopy. Multiple spectra (∼8-10) for each tissue site were measured with scanning times of 0.1 to 0.5 sec, which permits a rapid survey of large tissue areas. Raman spectra acquired in non-contact with esophageal tissues were automatically discarded online using Raman diagnostic software developed by our group^44^. A total of 1172 FP/HW esophageal Raman spectra (normal (n = 938) and ESCC (n = 234)) were successfully acquired *in vivo* from 101 tissue sites. Immediately after the tissue Raman acquisitions, each tissue site measured was biopsied and sent for histopathological examination by three senior gastrointestinal pathologists who were blinded to the Raman scans. The histopathology assessments serve as the gold standard to determine the diagnostic performance of the simultaneous FP/HW fiber-optic Raman technique for identifying ESCC from normal esophageal tissues.

### Statistical Analysis

Cohen’s κ statistics were calculated to examine the agreement among the pathologists^29^. The unpaired two-sided Student’s t-test was used to evaluate Raman spectral differences between ESCC and normal esophagus^15^. Mean-centering was performed prior to multivariate statistical analysis to remove common variance from the dataset of *in vivo* esophageal tissue Raman spectra. Partial least squares (PLS)- discriminant analysis (DA) was further applied for tissue diagnosis model development^45^. PLS-DA employs the fundamental principle of PCA but further rotates the components (latent variables (LVs)) by maximizing the covariance between the spectral variation and group affinity, so that the LVs explain the diagnostic relevant variations rather than the most prominent variations in the spectral dataset^45^. Leave-one-patient out, cross-validation was used to assess and optimize the PLS-DA model complexity, while reducing the risk of overfitting^45^. The overall discriminatory accuracy of the FP, HW, and integrated FP/HW diagnostic models were further evaluated through the use of the receiver operating characteristic (ROC) analysis^45^,^46^. The PLS-DA model developed was further applied to an independent validation dataset to evaluate the predictive diagnostic performance of the integrated FP/HW Raman spectroscopy technique for ESCC identification. The above multivariate statistical analysis was performed using in-house written scripts in the Matlab programming environment.

## Additional Information

**How to cite this article**: Wang, J. *et al.* Simultaneous fingerprint and high-wavenumber fiber-optic Raman spectroscopy improves *in vivo* diagnosis of esophageal squamous cell carcinoma at endoscopy. *Sci. Rep.* **5**, 12957; doi: 10.1038/srep12957 (2015).

## Supplementary Material

Supplementary Information
