# Supplementary material for: Simultaneous fingerprint and high-wavenumber fiber-optic Raman spectroscopy improves in vivo diagnosis of esophageal squamous cell carcinoma at endoscopy
Source: Sci Rep. 2015 Aug 5;5:12957. doi: 10.1038/srep12957 (PMC4525386; doi:10.1038/srep12957)

# Simultaneous fingerprint and high-wavenumber fiber-optic Raman spectroscopy improves *in vivo* diagnosis of esophageal squamous cell carcinoma at endoscopy

Jianfeng Wang[1], Kan Lin[1], Wei Zheng[1], Khek Yu Ho[2], Ming Teh[3],
Khay Guan Yeoh[2] & Zhiwei Huang[1]*

[1]Optical Bioimaging Laboratory, Department of Biomedical Engineering, Faculty of Engineering, National University of Singapore, Singapore 117576

[2]Department of Medicine, Yong Loo Lin School of Medicine, National University of Singapore and National University Health System, Singapore 119260

[3]Department of Pathology, Yong Loo Lin School of Medicine, National University of Singapore and National University Health System, Singapore 119074

**Subject Areas**: Translational Research; Raman spectroscopy

*Correspondence and requests for materials should be addressed to Z.H.
(biehzw@nus.edu.sg)

**Supplementary Fig. S1 |** Representative Raman spectrum of ESCC for diagnostic model development. The S/N ratio of Raman spectra is >10 by using the in vivo fiber-optic Raman technique developed. Prominent esophageal tissue Raman peaks with tentative assignments can be observed in the FP region, i.e., 853 (*v*(C-C) proteins), 1004 ($\nu_s$(C-C) ring breathing of phenylalanine), 1078 (*v*(C-C) of lipids), 1265 (amide III v(C-N) and δ(N-H) of proteins), 1302 ($CH_2$ twisting and wagging of lipids), 1335 ($CH_3CH_2$ twisting of proteins and nucleic acids), 1445 (δ($CH_2$) deformation of proteins and lipids), 1618 (v(C=C) of porphyrins), 1655 (amide I v(C=O) of proteins), and 1745 $cm^{-1}$ (v(C=O) of phospholipids). Intense Raman peaks are also observed in the HW region i.e., 2850 and 2885 $cm^{-1}$ (symmetric and asymmetric $CH_2$ stretching of lipids), 2940 $cm^{-1}$ ($CH_3$ stretching of proteins), ~3300 $cm^{-1}$ (amide A (NH stretching of proteins)) and the broad Raman band of water (OH stretching vibrations peaking at ~3250 and ~3400 $cm^{-1}$).

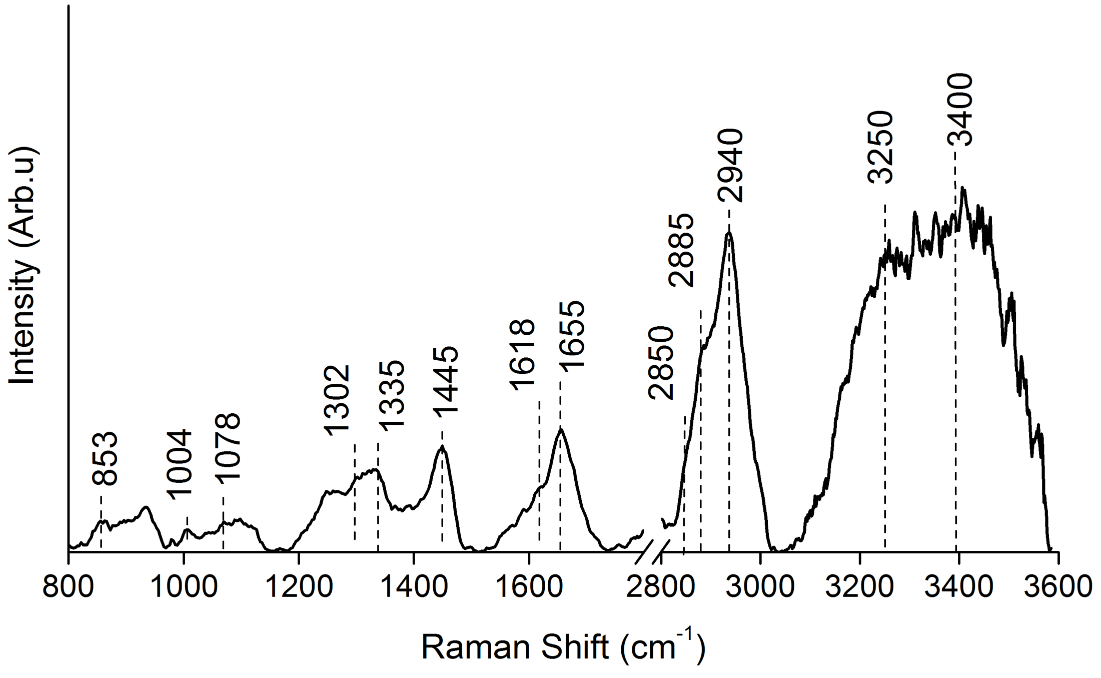

**Supplementary Fig. S2 |** The mean *in vivo* FP/HW Raman spectra ± 1 standard deviation (SD) of the testing dataset (20% of the total dataset) (normal (n=124); ESCC (n=110)) for independent validation.

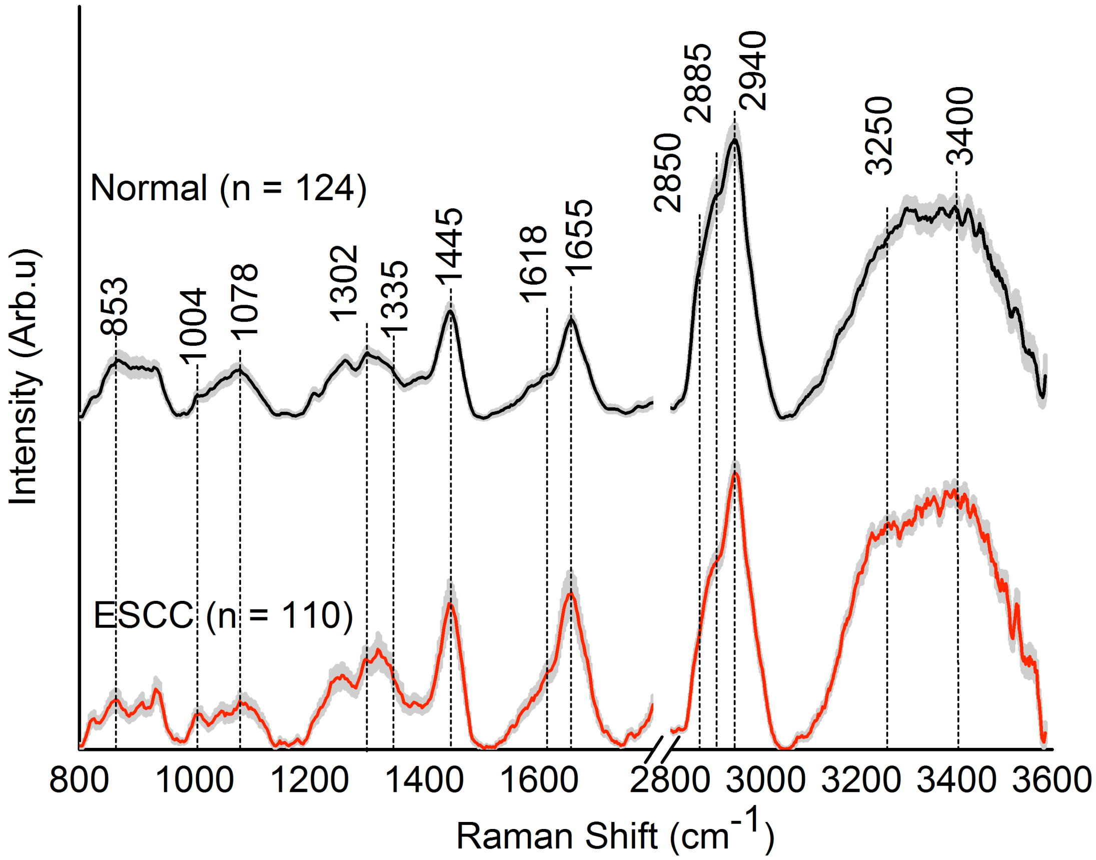

**Supplementary Fig. S3 |** The predictive probabilities of *in vivo* Raman spectra belonging to (i) normal esophagus (n=124), and (ii) ESCC (n=110) of the testing dataset (20% of the total dataset), using the diagnostic models developed with the FP, HW and the integrated FP/HW Raman techniques, respectively. (○) normal; (▲) ESCC.

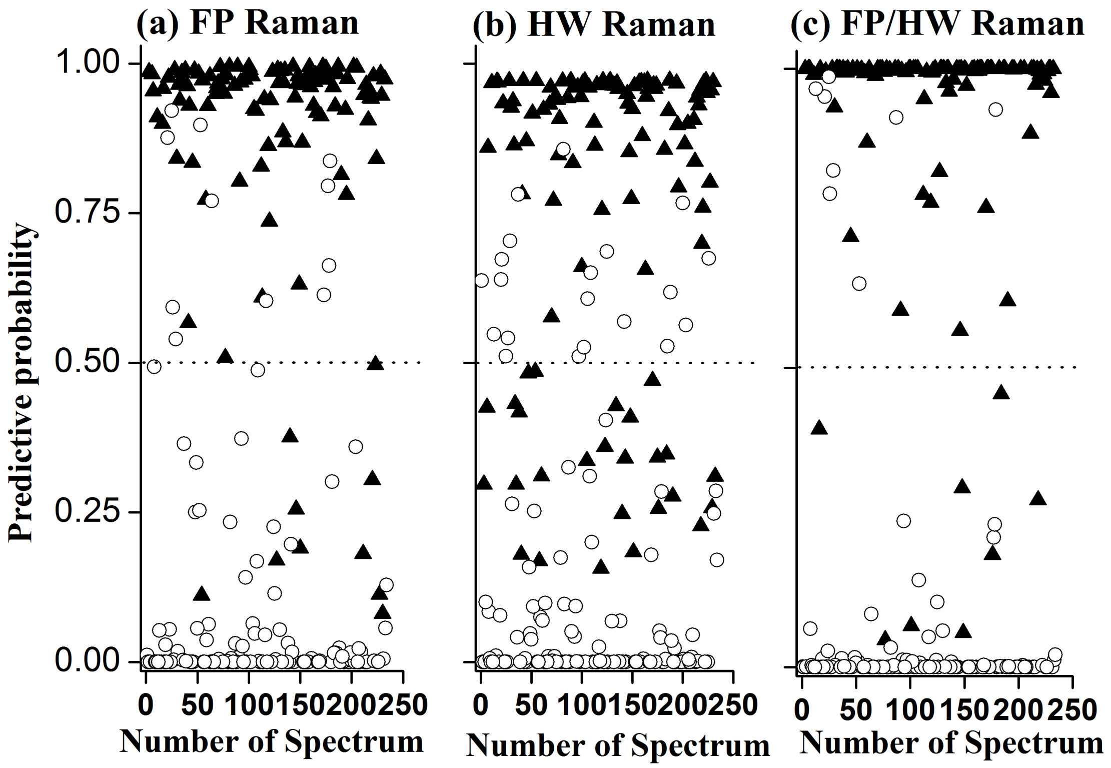

Supplement: Supplementary Information [file srep12957-s1.pdf]
